# Supplementary material for: CD229 (Ly9) a Novel Biomarker for B-Cell Malignancies and Multiple Myeloma
Source: Cancers (Basel). 2022 Apr 26;14(9):2154. doi: 10.3390/cancers14092154 (PMC9101303; doi:10.3390/cancers14092154)
Supplement: Supplementary file 1 [file cancers-14-02154-s001.zip › cancers-1653336-supplementary.pdf]

Supplementary Material: CD229 (Ly9) a Novel Biomarker for B-Cell Malignancies and Multiple Myeloma

Giovanna Roncador, Joan Puñet-Ortiz, Lorena Maestre, Luis Gerardo Rodríguez-Lobato, Scherezade Jiménez, Ana Isabel Reyes-García, Álvaro García-González, Juan F. García, Miguel Ángel Piris, Santiago Montes-Moreno, Manuel Rodríguez-Justo, Mari-Pau Mena, Carlos Fernández de Larrea and Pablo Engel

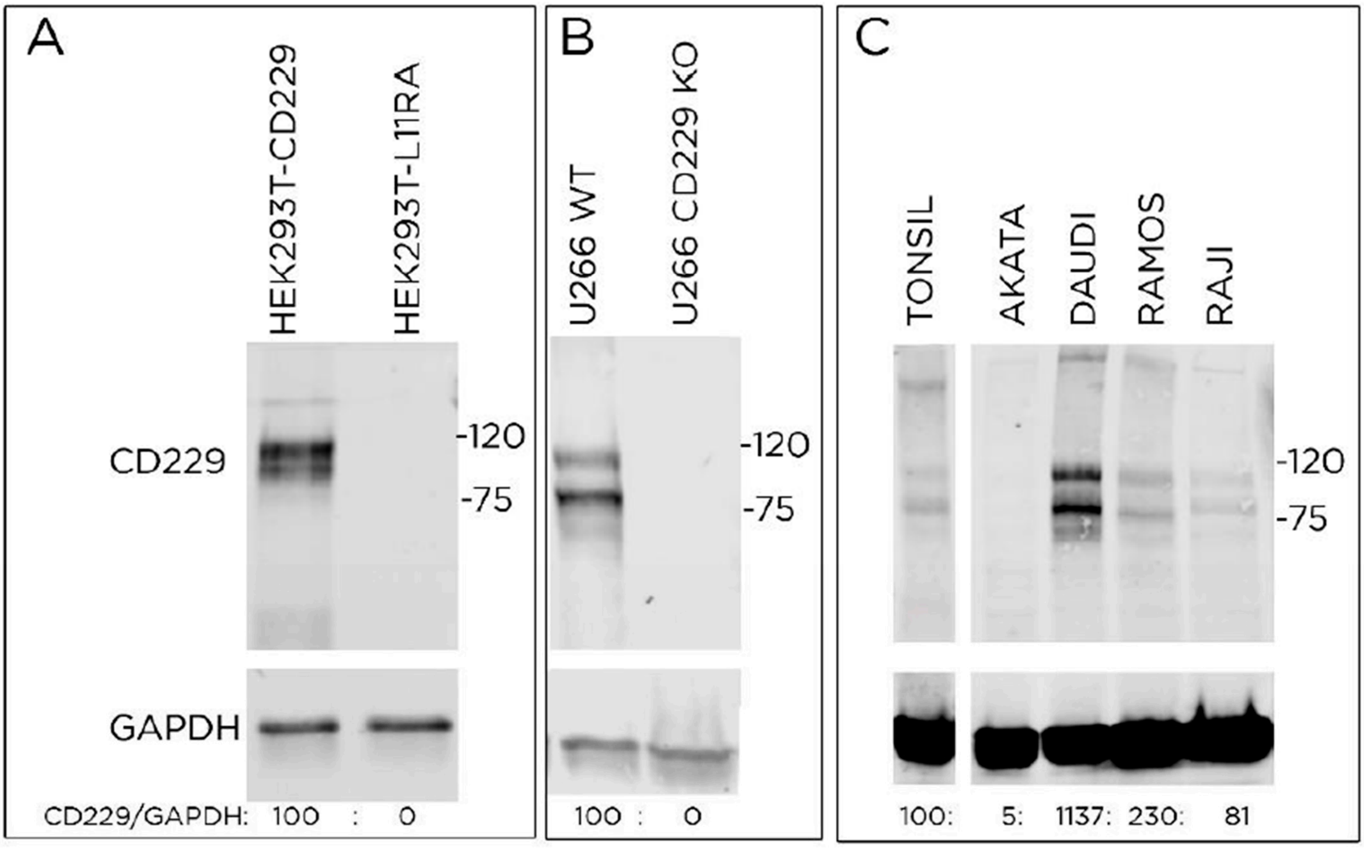

Figure S1. Western blot analysis of PIZCU426A mAb reactivity. (**A**) CD229 transfected HEK293T versus HEK293T cells transfected with an unrelated protein (IL11RA); (**B**) U266 WT versus U266 CD229 deficient (KO) cells and (**C**) tonsil and CD229 positive B cell lines (DAUDI, RAMOS and RAJI) and negative cell line AKATA. Densitometry reading/intensity ratio of each band are included. Uncut blots are available in Figure S4.

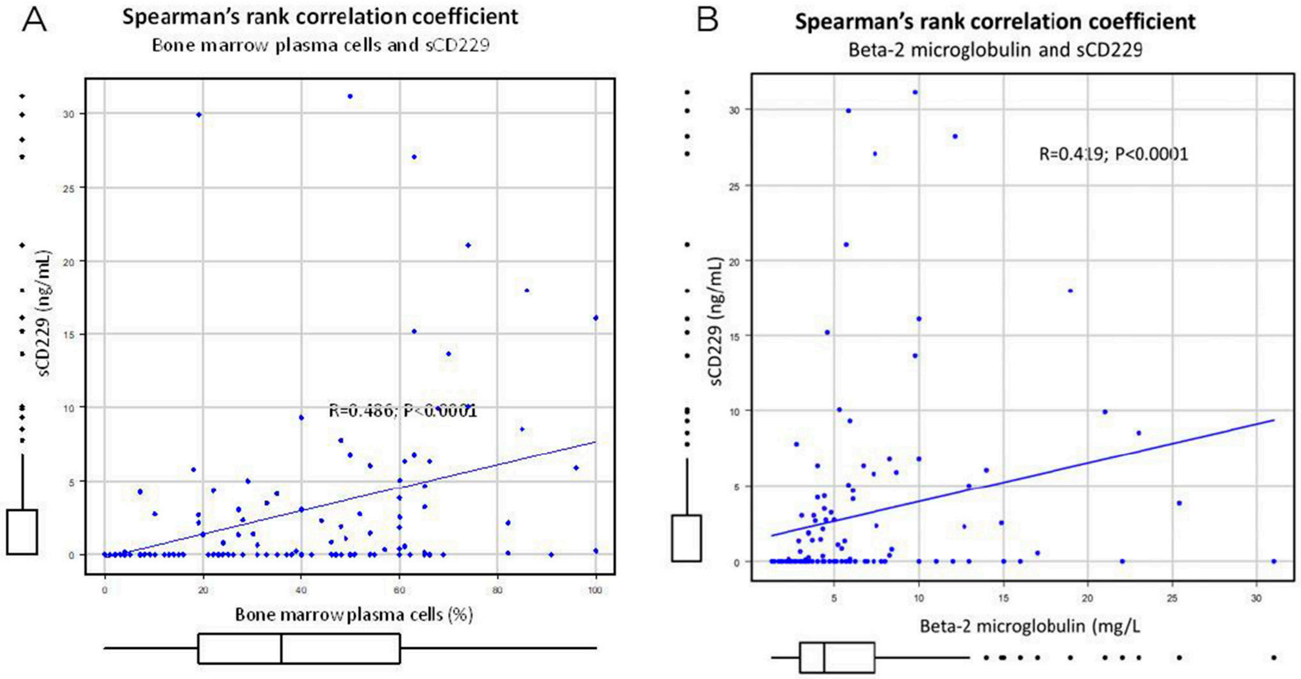

Figure S2. Scatter plot showing correlation between the percentage of bone marrow plasma cells (**A**) serum β2 microglobulin (**B**) and sCD229 in 122 newly diagnosed multiple myeloma patients.

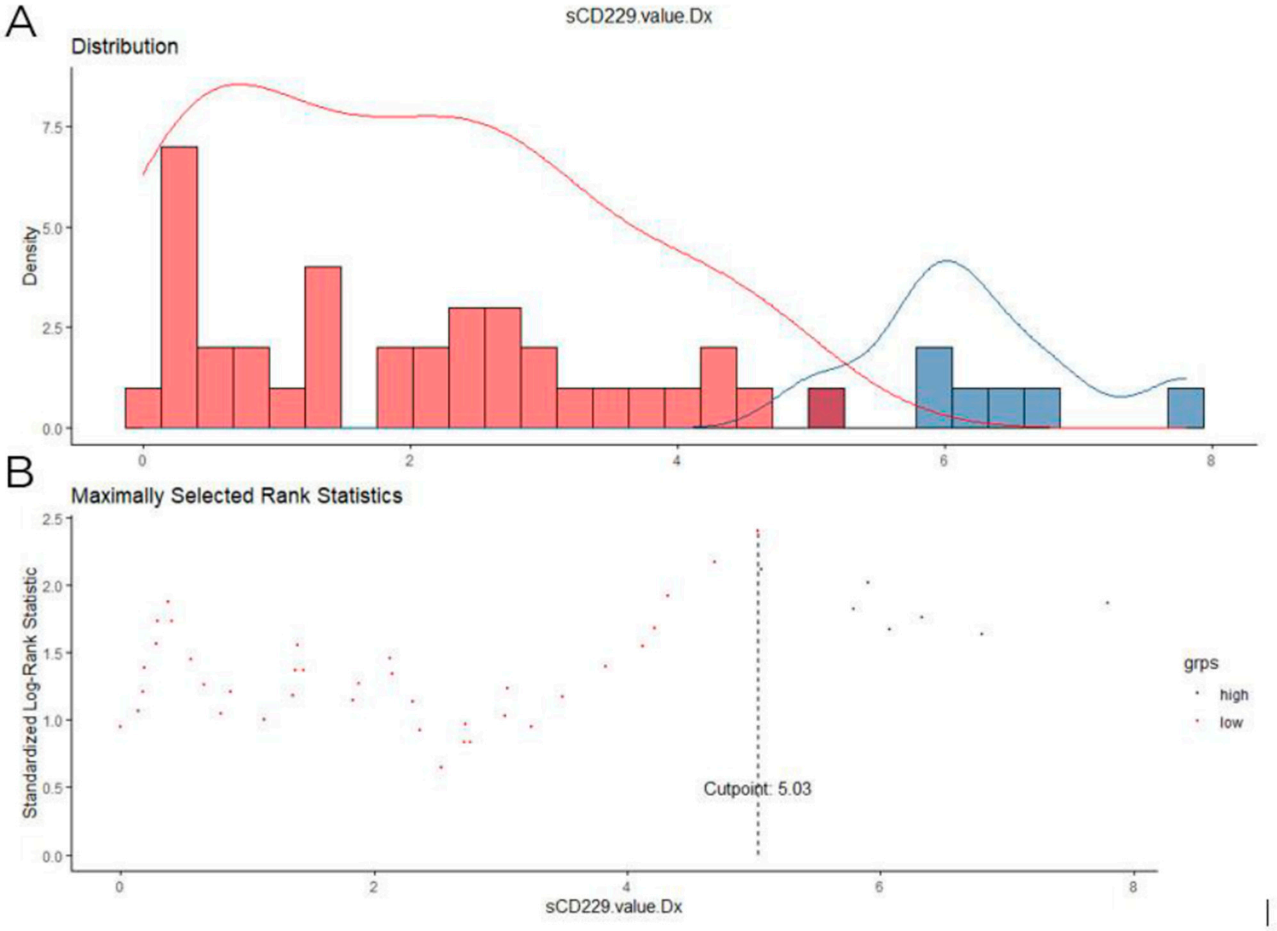

Figure S3. Identification of the best cut-off value of the soluble CD229 to predict overall survival using the maximally selected rank statistics from the "maxstat" and "survminer" R packages. The

x-axis represents the value of sCD229 (**A** and **B**), while the y-axis represents the number of samples (**A**) and the results of the log rank test for overall survival (**B**).

Table S1. Multivariate analysis for PFS and OS.

Multivariate analysis for PFS			
**Variable**	**HR**	**95% CI**	***P***
Age	1.03	1.01–1.06	0.003
Hemoglobin	1.00	0.99–1.02	0.67
Albumin	0.99	0.95–1.03	0.60
β2-microglobulin	1.06	1.02–1.10	0.003
sCD229 > 5.0 ng/mL	1.56	0.92–2.67	0.10
**Multivariate analysis for OS**			
Age	1.05	1.02–1.08	0.002
Hemoglobin	1.002	0.98–1.02	0.88
Albumin	0.99	0.93–1.05	0.68
β2-microglobulin	1.09	1.04–1.15	<0.001
sCD229 > 5.0 ng/mL	1.62	0.82–3.22	0.16

CI: confidence interval; HR: hazard ratio.

Table S2. Antibodies used in the study.

Molecule	Antibody Type	Clone Name	Source
CD20	Recombinant rabbit mono-clonal	SP32	Abcam
CD229	Rat monoclonal	PIZCU426A	CNIO
CD229	Mouse monoclonal	1.25	UB
CD229	Mouse monoclonal	1.84	UB
CD3	Rabbit polyclonal	IR503	FLEX
GAPDH	Mouse monoclonal	FF26A	CNIO
PD1	Mouse monoclonal	NAT105C	CNIO

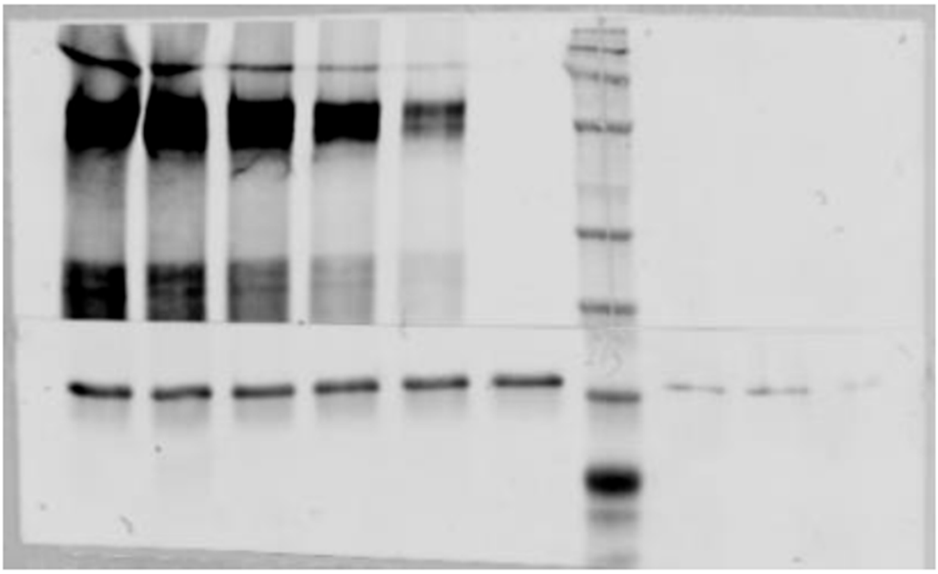

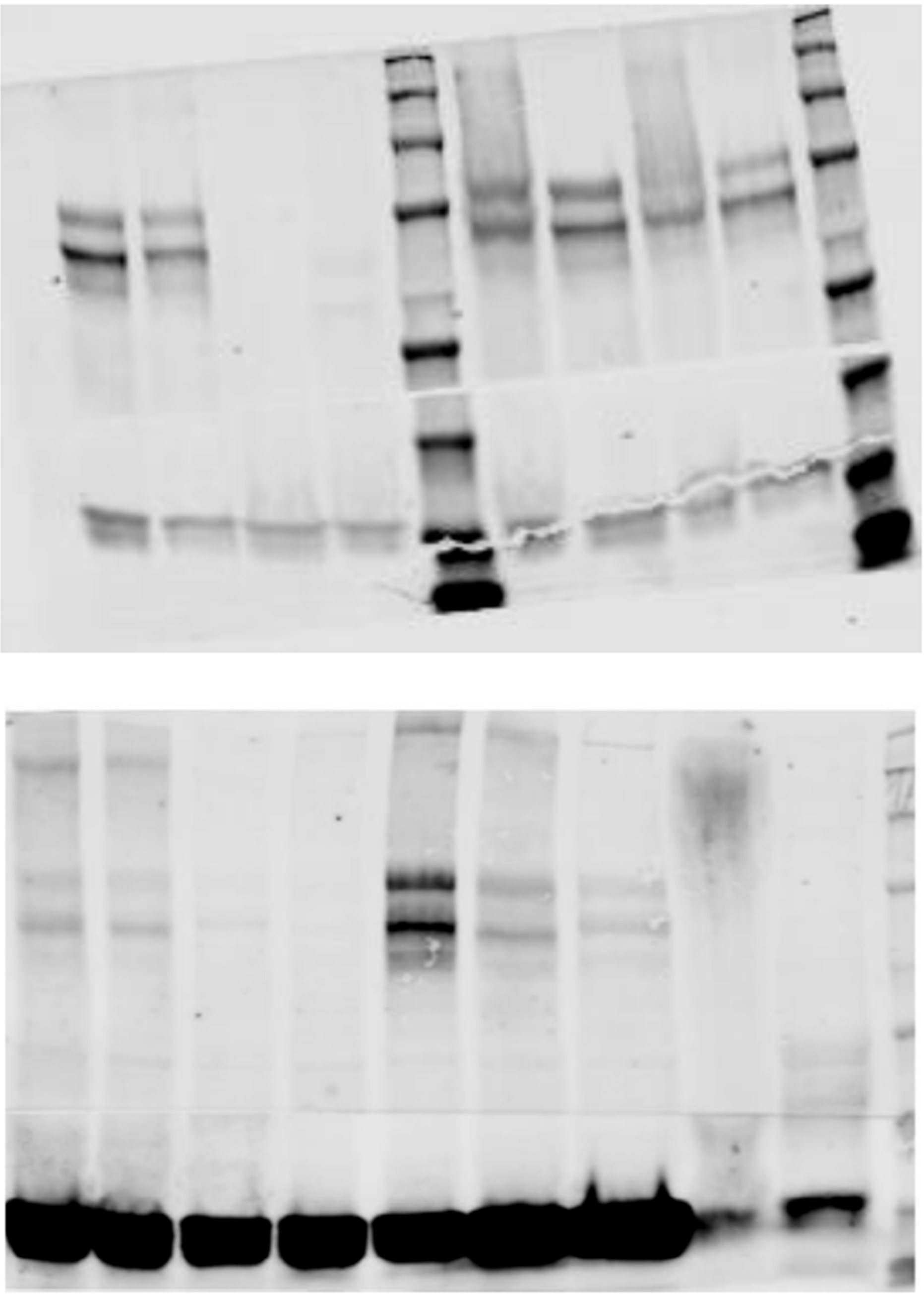

Figure S4. Raw images of WB from Figure S1.

Publisher's Note: MDPI stays neutral with regard to jurisdictional claims in published maps and institutional affiliations.

© 2022 by the authors. Licensee MDPI, Basel, Switzerland. This article is an open access article distributed under the terms and conditions of the Creative Commons Attribution (CC BY) license (http://creativecommons.org/licenses/by/4.0/).
